# Higher visit-to-visit total cholesterol variability is associated with lower cognitive function among middle-aged and elderly Chinese men

**DOI:** 10.1038/s41598-020-72601-7

**Published:** 2020-09-23

**Authors:** Jianian Hua, Yanan Qiao, Chaofu Ke, Yueping Shen

**Affiliations:** 1 Department of Neurology, The First Affiliated Hospital of Soochow University, Suzhou, Jiangsu People’s Republic of China; 2 Medical College of Soochow University, Suzhou, 215123 People’s Republic of China; 3 Department of Epidemiology and Biostatistics, School of Public Heath, Medical College of Soochow University, 199 Renai Road, Suzhou, 215123 People’s Republic of China

**Keywords:** Alzheimer's disease, Cognitive ageing, Cognitive neuroscience, Risk factors

## Abstract

To examine the prospective associations between total cholesterol (TC) variability and cognitive function in a large sample of Chinese participants aged 45 years and above. A total of 6,377 people who participated in the China Health and Retirement Longitudinal Study (CHARLS) were included. TC variability was defined as the intra-individual standard deviation over two blood tests in CHARLS 2011 and 2015 (Wave 1 and Wave 3). Cognitive function was assessed by a global cognition score, which included three tests: episodic memory, figure drawing and Telephone Interview of Cognitive Status (TICS). Multivariate linear regression models (MRLMs) and generalized estimating equation (GEE) were used to investigate associations between TC variability and cognitive scores. After adjusting for potential confounders, male participants with higher visit-to-visit TC variability showed lower global cognition scores (*β* = − 0.71, *P* < 0.001). After further adjustment for baseline cognition, the association remained statistically significant (*β* = − 0.68, *P* < 0.001). The domains with declines were focused on episodic memory (*β* = − 0.22, *P* = 0.026) and TICS (*β* = − 0.44, *P* = 0.004). However, these associations were not found in women (*β* = − 0.10, *P* = 0.623). For men, the rates of decline in global cognition increased by 0.14 (*β* = − 0.14, *P* = 0.009) units per year while TC variability increased by 1 mmol/L. For males, higher visit-to-visit TC variability correlated with lower cognitive function and an increased rate of decreases in memory. More attention should be paid to cognitive decline in males with high TC variability, and particularly, on decreases in memory, calculation, attention and orientation.

## Introduction

Over 90 years ago, Walter Cannon coined the term “homeostatis”^1^, which hypothesized that the variability in constituents in the body is associated with abnormalities in human’s function. Under the guidance of this theory, recent studies have found that increased variability in lipid levels was related to high risk of mortality, atrial fibrillation, or myocardial infarction^2–5^. In recent years, patients at high cardiovascular risk receive intensive lipid-lowering therapy, leading to higher cholesterol variability. The effect of lipid-lowering therapy on neurocognitive function is disputable. Some evidence has indicated memory loss, while other studies have suggested beneficial outcomes. In contrast with TC concentration which was used most^6–8^, TC variability was a new and seldom studied marker. To date, a total of 3 studies have found that cholesterol variability was associated with dementia or cognitive function^9–12^. *RAJ. Smit 2016 *showed an association between high variability in low density lipoprotein (LDL) levels and low cognitive function. *A. Solomon 2007* reported that changes in serum total cholesterol (TC) was associated with dementia, while *HS. Hung 2019* linked high TC variability to high risk of dementia. Nevertheless, the longitudinal associations with cognitive function have yet to be examined. More importantly, there was no knowledge about the cholesterol variability associated with cognitive decline in China. Therefore, we used a national representative database, the China Health and Retirement Longitudinal Study (CHARLS), to investigate cognitive function among people with high variability in cholesterol levels.

## Materials and methods

### Study sample

The China Health and Retirement Longitudinal Study (CHARLS) aimed to collect nationally representative sample of Chinese residents. The Wave 1 of CHARLS included about 17,000 individuals from 150 counties/districts and 450 villages/resident committees. The subjects of the study were Chinese citizens aged 45 years and above^13^. Baseline data (Wave 1) were collected between June 2011 and March 2012. In addition, the survey was conducted and followed up every 2 years.

**Figure 1:**
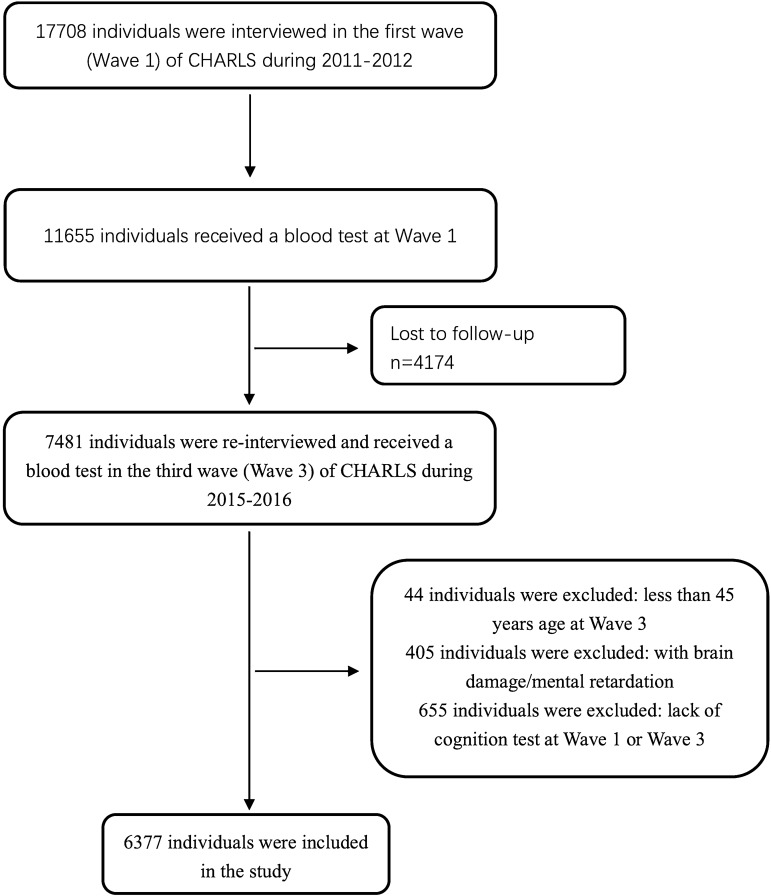
Flow chart of the sample selection and exclusion criteria.

In the CHARLS, a total number of 7,481 participants received blood testing twice, in 2011 (Wave 1) and 2015 (Wave 3). Of the 7,481 individuals, 44 individuals under 45 years old were excluded, 405 individuals with a history of brain damage or mental retardation were excluded, and 655 individuals without the cognitive tests were excluded. Eventually, 6,377 individuals were included in this study. The selection diagram and criteria for exclusion were provided in Fig. 1.

### Assessment of cognition

Cognitive function was evaluated through three kinds of tests: episodic memory, figure drawing, and Telephone Interview of Cognitive Status (TICS). Serving as the primary outcome, the global cognition score was the sum of the three test scores. The global cognition score ranges from 0 to 21. The average score of all participants was about 10. The cognitive function was tested in Wave 1 and Wave 3, by face-to-face interview. Cognitive function in Wave 3 was used as the outcome.

The episodic memory test reflected individuals’ function of memory. In the test, the participants were asked to memorize and recall the words immediately (immediate recall) and 5 min later (delayed recall) after interviewers read 10 Chinese nouns to them^14^. The episodic memory score was the average score of the immediate recall and delayed recall tests and could range from 0 to 10. The figure drawing test examined the visuospatial function. In the figure-drawing test, the participants were shown a picture and asked to redraw it. If the participant failed, the figure-drawing score was 0, and if the participant succeeded, the score was 1. The TICS test reflected function of attention, calculation and attention. This test was based on selected questions from the TICS battery in mini-mental state examination (MMSE), and was reliable for research on cognition^15,16^. In this test, the participants were asked to repeatedly subtract 7 from 100 and to identify the date, season, and day of the week. The TICS scores could range from 0 to 10.

### Assessment of TC variability

Blood samples of all the participants were collected after an overnight fast by trained staff in the Chinese Center for Disease Control and Prevention (Chinese CDC). Venous blood was turned into plasma and buffy coat and was immediately frozen and stored at − 20 °C; the samples were transported to the Chinese CDC in Beijing within 2 weeks, where they were placed in a deep freezer and stored at − 80 °C until quantified at Capital Medical University (CMU) laboratory^13^. In the statistical analyses, we converted the units from mg/dL to mmol/L.

The CHARLS had three waves, however, the blood testing was only carried out twice, in Wave 1 and Wave 3. The visit-to-visit variability in TC (TC variability) was calculated by intra-individual standard deviations (SDs) across the two tests^10^.

Because the level of most people’s TC variability was small, and there were no guidelines for abnormal levels of variability, we chose the SD as cut-off points for a simple and clear categorization^12^. Based on the SD in the TC variability, we divided men into 4 groups: ≤ 0.306 mmol/L, 0.306–0.612 mmol/L, 0.612–0.918 mmol/L, > 0.918 mmol/L as Q1, Q2, Q3 and Q4, respectively. In addition, women were grouped as Q1 (≤ 0.278 mmol/L), Q2 (0.278–0.556 mmol/L), Q3 (0.556–0.834 mmol/L) and Q4 (> 0.834 mmol/L).

### Potential confounders

Because of socioeconomic status, elderly Chinese females have a lower cognitive function than men^17^. Some studies have reported the gender differences when taking dementia as the outcome^18^. Thus, we conducted our research on men and women separately.

The potential confounders consisted of health factors and diseases recorded in CHARLS Wave 1. They are all known to be associated with blood cholesterol concentration and cognitive function. The average of TC levels in Wave 1 and Wave 3 was adjusted in all models. In Tables 3 and 4, Model 1 was the minimally adjusted model. It included age, education, marital status, residential area, mean TC concentration, and BMI. Model 2 was the fully adjusted model. It further included smoking, drinking, depression, lipid-lowering therapy, and six comorbidities.

We analysed the effect of drugs on cognitive function by using “lipid-lowering therapy” as a categorical variable (yes or no). Depression was classified as “yes” and “no”, using the 10-item Center for Epidemiologic Studies Short Depression Scale (CES-D-10). This score can range from 0 to 30, and the cut-off point for depression was 12^19^. The comorbidities included hypertension, dyslipidaemia, diabetes, cancer or tumour, heart problems and stroke, which were assessed in Wave 1.

### Statistical analysis

Demographic characteristics were presented as the mean ± SD or frequency (percentage). Associations between participants’ characteristics and TC variability were examined using ANOVA or the Pearson χ^2^ test. The linear correlations between TC variability and cognitive function in Wave 3 were calculated using multivariate linear regression models (MRLMs) after adjustment for potential confounders. In Table 2, TC variability was defined as a categorical variable. Individual in Q1 group had the highest mean global cognition score. We compared Q2, Q3, Q4 with Q1 separately. We also used generalized estimating equation (GEE) to extend the linear model for further analysis of the longitudinal associations. In GEE, the interaction of the time variable with TC variability was conducted to examine whether rates of cognitive decline varied by levels of TC variability. All data were analysed by using SAS version 9.4 (SAS Institute Inc., Cary, North Carolina, USA), and *P* < 0.05 was defined as the significance level.

### Ethics statement

Each participant included in this study signed a written informed consent form before taking the survey. Ethics approval for the data collection in the CHARLS was obtained from the Biomedical Ethics Review Committee of Peking University (IRB00001052-11015). We confirm that all methods were performed in accordance with the relevant guidelines and regulations.

## Results

### Demographic and health characteristics of the study population in Wave 1 among the different TC groups

The mean ± SD of TC variability among all people was 0.32 ± 0.29 mmol/L. At baseline, the mean age of the participants was 58.4 ± 8.7 years; 45.4% of the participants were male; 74% of them finished their education in primary school; and 79.7% of the participants were from rural areas.

Higher TC variability was associated with higher mean TC concentrations (*P* < 0.001). Among all the participants, more than 5% were receiving lipid-lowering therapy for dyslipidaemia. In addition, in the groups with higher TC variability, more individuals were receiving therapy (*P* < 0.001). Participants with higher TC variability tended to live in urban area (*P* = 0.004) and drink (*P* = 0.014). The subjects with higher TC variability were more likely to have depression (*P* = 0.015), hypertension (*P* < 0.001) and dyslipidaemia (*P* = 0.025). We found no associations between TC variability and age, gender, education, marital status, BMI, smoking, and history of diabetes mellitus, heart disease and stroke (Table 1).

**Table 1:** Demographic and health characteristics of the study population in Wave 1 among the different TC groups.

	Q1 (n = 3,607)	Q2 (n = 1,850)	Q3 (n = 605)	Q4 (n = 315)	*P* value
**Continuous variables**					
Age, years	58.4 ± 8.9	58.3 ± 8.6	58.8 ± 8.5	58.8 ± 8.2	0.418
Mean TC^a^, mmol/L	4.8 ± 0.8	4.9 ± 0.8	5.1 ± 0.9	5.4 ± 1.1	< 0.001
Categorical variables, n (%)					
Male	1647 (45.7)	829 (44.8)	266 (44.0)	150 (47.8)	0.664
**Education**					0.579
Illiterate	905 (25.1)	494 (26.7)	173 (28.6)	91 (28.9)	
Primary school	1562 (43.3)	789 (42.3)	258 (42.7)	135 (42.9)	
Middle school	771 (21.4)	383 (21.2)	113 (18.7)	60 (19.1)	
High school and above	368 (10.2)	174 (9.4)	60 (9.9)	29 (9.2)	
**Marital status**					0.743
Married	3,244 (89.9)	1671 (90.3)	550 (90.9)	281 (91.1)	
Other status	363 (10.1)	179 (9.7)	55 (9.1)	38 (8.9)	
**Residential area**					0.004
Urban	612 (16.6)	299 (15.8)	88 (13.5)	75 (23.0)	
Rural	3,076 (83.4)	1,590 (84.2)	537 (85.9)	251 (77.0)	
**Depression**					0.015
Yes	264 (7.1)	108 (5.7)	58 (9.3)	26 (8.0)	
No	3,431 (92.9)	1784 (94.3)	567 (90.7)	300 (92.0)	
**BMI (kg/m**^**2**^**)**					0.115
< 18.5	561 (15.2)	264 (14.0)	87 (13.4)	50 (15.3)	
18.5–28	2,742 (74.2)	1,392 (736.)	464 (74.2)	227 (69.6)	
> 28	392 (10.6)	236 (12.5)	74 (11.8)	49 (15.0)	
Current smoker	1,408 (38.1)	734 (38.8)	229 (36.6)	136 (41.7)	0.460
Current drinker	873 (23.6)	501 (26.5)	166 (26.6)	97 (29.8)	0.014
Hypertension	872 (23.6)	474 (25.1)	192 (30.7)	105 (42.2)	< 0.001
Dyslipidaemia	346 (9.4)	177 (9.4)	76 (12.2)	43 (13.2)	0.025
Diabetes mellitus	192 (5.2)	115 (6.1)	49 (7.8)	18 (5.5)	0.056
History of heart disease	423 (11.5)	236 (12.5)	82 (13.1)	45 (13.8)	0.361
History of stroke	69 (1.9)	32 (1.7)	12 (1.9)	9 (2.8)	0.626
Lipid-lowering therapy	216 (5.9)	139 (7.4)	70 (11.2)	52 (15.9)	< 0.001

Four groups were categorized by the SD in TC variability (SD = 0.306 mmol/L): Q1: ≤ 0.306 mmol/L, Q2: 0.306–0,612 mmol/L, Q3: 0.612–0.918 mmol/L, Q4: > 0.918 mmol/L.

### Mean cognition scores in the four categories of TC variability in Wave 3

We divided all individuals by gender, into 4 groups (Q1 to Q4) using the same method shown in Table 1. The participants with higher TC variability had lower cognitive function. After defining the dummy variables for the TC variability, *P* values for each dummy variable were calculated with a linear regression model after adjusting for age. In men, although the mean cognition scores decreased in each group, only the third group showed significance (*P* < 0.05). In addition, in women, there were no associations in these groups (Table 2).

**Table 2:** Mean cognition scores in the four categories of TC variability in Wave 3.

Sex	TC variability	N	Global cognition		Episodic memory		Figure drawing		TICS	
			X ± s	*P* value	X ± s	*P* value	X ± s	*P* value	X ± s	*P* value
Male	Q1 (≤ 0.306 mmol/L)	1703	11.14 ± 3.91	Ref	3.32 ± 1.81	Ref	0.71 ± 0.45	Ref	7.11 ± 2.60	Ref
	Q2 (0.306–0,612 mmol/L)	829	11.05 ± 3.84	0.409	3.30 ± 1.76	0.682	0.71 ± 0.45	0.683	7.04 ± 2.60	0.472
	Q3 (0.612–0.918 mmol/L)	243	10.69 ± 4.10	0.009	3.26 ± 1.72	0.250	0.66 ± 0.47	0.058	6.77 ± 2.78	0.006
	Q4 (> 0.918 mmol/L)	122	10.88 ± 3.96	0.155	3.20 ± 1.64	0.111	0.66 ± 0.47	0.210	7.02 ± 2.70	0.189
Female	Q1 (≤ 0.278 mmol/L)	1899	9.59 ± 4.46	Ref	3.30 ± 1.86	Ref	0.54 ± 0.50	Ref	5.76 ± 2.97	Ref
	Q2 (0.278–0.556 mmol/L)	1,040	9.52 ± 4.57	0.728	3.27 ± 1.90	0.790	0.51 ± 0.50	0.078	5.75 ± 3.02	0.766
	Q3 (0.556–0.834 mmol/L)	361	8.93 ± 4.53	0.542	3.06 ± 1.84	0.655	0.48 ± 0.50	0.844	5.40 ± 3.09	0.591
	Q4 (> 0.834 mmol/L)	185	9.12 ± 4.93	0.427	3.13 ± 1.98	0.697	0.54 ± 0.50	0.281	5.45 ± 3.19	0.413

Adjusted for age, education, mean TC, marital status, residential area, BMI, smoking, drinking, depression, lipid-lowering therapy, hypertension, dyslipidaemia, diabetes mellitus, stroke, heart disease, and cancer.

### Associations between TC variability and cognitive function (dependent variable) in Wave 3

For males, higher TC variability was significantly associated with lower global cognition scores in Wave 3 (*P* < 0.001 for both model 1 and model 2). The domains showing decreased scores were episodic memory (*P* = 0.023 for model 1 and *P* = 0.026 for model 2) and TICS (*P* = 0.004 for both model 1 and model 2). There was no association in the figure drawing test (*P* = 0.053 for model 1 and *P* = 0.054 for model 2). For females, we found no evidence for an association between TC variability and cognition scores (Table 3).

**Table 3:** Associations between TC variability and cognitive function (dependent variable) in Wave 3.

	Model 1		Model 2	
	β (95% CI)	*P* value	β (95% CI)	*P* value
**Male**				
Global cognition	− 0.71 (− 0.92, − 0.50)	< 0.001	− 0.71 (− 0.92, − 0.50)	< 0.001
Episodic memory	− 0.22 (− 0.32, − 0.12)	0.023	− 0.22 (− 0.31, − 0.12)	0.026
Figure drawing	− 0.05 (− 0.08 to − 0.03)	0.053	− 0.05 (− 0.08 to − 0.02)	0.054
TICS	− 0.43 (− 0.59, − 0.29)	0.004	− 0.44 (− 0.59, − 0.29)	0.004
**Female**				
Global cognition	− 0.07 (− 0.29, 0.15)	0.750	− 0.10 (− 0.32, 0.11)	0.623
Episodic memory	− 0.00 (− 0.10, 0.10)	0.982	0.01 (− 0.09, 0.11)	0.903
Figure drawing	0.01 (− 0.03, 0.02)	0.816	0.01 (− 0.02, 0.03)	0.800
TICS	− 0.07 (− 0.23 to 0.08)	0.639	− 0.13 (− 0.28, 0.03)	0.423

Model 1: adjusted for age, education, mean TC, marital status, residential area, and BMI.

Model 2: adjusted for Model 1 + smoking, drinking, depression, lipid-lowering therapy, hypertension, dyslipidaemia, diabetes mellitus, stroke, heart disease, and cancer.

Using multiple linear regression model, the adjusted unstandardized regression coefficients and *P* values were calculated with TC variability (mmol/L) used as a continuous measurement.

### Associations between TC variability and cognitive function in Wave 3 after adjusting for baseline cognition

These associations were essentially unchanged in the model that added baseline (Wave 1) cognition as a covariate. For males, high TC variability was significantly associated with lower global cognition scores (*P* < 0.001 for both model 1 and model 2). The domains showing decreased score were episodic memory (*P* = 0.012 for model 1 and *P* = 0.014 for model 2) and TICS (*P* = 0.005 for model 1 and *P* = 0.003 for model 2). This association was not seen in the figure drawing test (*P* = 0.067 for model 1 and *P* = 0.064 model 2). For females, we found no evidence for associations between TC variability and cognition scores (Table 4).

**Table 4:** Associations between TC variability and cognitive function in Wave 3 after adjusting for baseline cognition.

	Model 1		Model 2	
	β (95% CI)	*P* value	β (95% CI)	*P* value
**Male**				
Global cognition	− 0.67 (− 0.86, − 0.48)	< 0.001	− 0.68 (− 0.87, − 0.49)	< 0.001
Episodic memory	− 0.23 (− 0.33, − 0.14)	0.012	− 0.23 (− 0.32, − 0.14)	0.014
Figure drawing	− 0.05 (− 0.08 to -0.02)	0.067	− 0.05 (− 0.08, − 0.03)	0.064
TICS	− 0.39 (− 0.53, − 0.25)	0.005	− 0.44 (− 0.59, − 0.29)	0.003
**Female**				
Global cognition	− 0.10 (− 0.31, 0.08)	0.571	− 0.13 (− 0.33, 0.05)	0.476
Episodic memory	− 0.01 (0.10, 0.08)	0.903	0.00 (− 0.10, 0.09)	0.963
Figure drawing	0.01 (− 0.01 to 0.03)	0.719	0.01 (− 0.02, 0,04)	0.724
TICS	− 0.10 (− 0.24 to 0.04)	0.465	− 0.14 (− 0.31, − 0.03)	0.298

Model 1: adjusted for baseline cognition, age, mean TC, education, marital status, residential area, and BMI.

Model 2: adjusted for Model 1 + smoking, drinking, depression, lipid-lowering therapy, hypertension, dyslipidaemia, diabetes mellitus, stroke, heart disease, and cancer.

Using multiple linear regression model, the adjusted unstandardized regression coefficients and *P* values were calculated with TC variability (mmol/L) used as a continuous measure.

### Longitudinal cognitive changes by TC variability using GEE model*

Among males, the TC variability-by-time interaction was statistically significant (*β* = -0.14, *P* = 0.009). As TC variability increased 1 mmol/L in males, the decline rate in global cognition increased 0.14 units (global cognition score) per year (Table 5). The specific domain was episodic memory (*β* = − 0.06, *P* = 0.031). Figure drawing and TICS were not significant.

**Table 5:** Longitudinal cognitive changes by TC variability using GEE model.

Cognition test	Intercept β (95% CI)	*P* value	Time β (95% CI)	*P* value	TC variability × time β (95% CI)	*P* value
**Male**						
Global cognition	10.34 (9.76, 10.92)	< 0.001	− 0.03 (− 0.06, − 0.01)	0.174	− 0.14 (− 0.19, − 0.09)	0.009
Episodic memory	4.50 (4.23, 4.77)	< 0.001	0.04 (0.02, 0.05)	0.007	− 0.06 (− 0.08, − 0.04)	0.031
Figure drawing	0.62 (0.56, 0.69)	< 0.001	− 0.01(− 0.01, 0.00)	0.044	− 0.02 (− 0.02, 0.00)	0.238
TICS	5.22 (4.82, 5.62)	< 0.001	− 0.06 (− 0.08, − 0.05)	< 0.001	− 0.07 (− 0.11, − 0.03)	0.081

Adjusted for all potential confounders.

## Discussion

We examined the longitudinal relationship between visit-to-visit total cholesterol (TC) variability and cognitive function among 6,377 middle-aged and elderly Chinese participants. It was identified that higher TC variability was associated with lower global cognition scores and higher rates of decline in global cognition scores over a period of 4 years in men, even after adjustments for mean TC variability at baseline and other potential confounders. For cognitive scores in Wave 3, the main affected domains were episodic memory and TICS. For rates of cognitive decline, the main affected domain was episodic memory. However, we found no associations in women.

Our results were similar to those from a previous study^10^, which reported that participants with high cholesterol variability had lower cognitive function. Nevertheless, our study had three differences. First, using MRLMs and GEE, we examined the longitudinal relationship that higher TC variability was associated with faster rates of decline over a period of 4 years. Second, we found this association did not exist in women. Third, we provided additional evidence for previous findings that the cognitive degeneration domains were episodic memory and TICS which represented abilities in memory, calculation and orientation. Individuals’ visuospatial ability, reflected by “figure drawing”, also declined but was not significant. Last but not least, in the past 3 years, some studies have reported other effects of TC variability or LDL variability^2–5^. Together with our study, there are indications that cholesterol variability could be a target for further research.

The association was not significant between LDL variability and cognition in our study, although the relationship was also negative (Supplementary Material 3). In serum, TC is mainly composed of LDL. LDL is sometimes called “bad” cholesterol, for it moves cholesterol around the blood and deposits it on the artery walls^20^. Therefore, compared with TC, LDL is viewed as a more “precise” target in the treatment of atherosclerosis cardiovascular disease^21^. In many studies which learned cholesterol, if TC made significance, LDL would make significance. However, we did not find LDL variability was associated with lower cognition. One previous study also reported, variability in TC, instead of LDL, was associated with lower cognition^9^. Limited by current studies, we could not explain this phenomenon.

There are several explanations for our findings. Both animal^22^ and clinical^23^ studies have demonstrated that lipid-lowering therapy could reconstruct carotid atherosclerotic plaques and make the plaques unstable. Thus, cholesterol variability might break the plaque into tiny pieces, thereby increasing the risk of subclinical cerebrovascular damage^24,25^. Furthermore, studies have reported that high LDL variability was associated with lower cerebral blood flow and endothelial dysfunction, which are linked to poor cognition^26,27^.

Whether lipid-lowering treatment affects cognition remains unclear^28–32^. Due to the sample size and cross-sectional nature of this study, we could not find strong evidence that lipid-lowering treatment led to cognitive change (Supplementary Material 1). In our study, receiving lipid-lowering therapy might be the result of dyslipidaemia and vascular diseases, which have been associated with lower cognition. Nevertheless, our findings highlight the need for concern about TC variability among people receiving lipid-lowering treatment. Currently, an expanding extent of lipid-lowering treatment has been recommended by doctors to prevent people at high risk from cardiovascular disease. A meta-analysis showed more intensive lipid-lowering therapy could reduce cardiovascular mortality^33^. In China, the proprotein convertase subtilisin-kexin type 9 (PCSK9) monoclonal antibodies have been in clinical use since 2019, which are known to produce high TC variability^34^. On the basis of our results, it is imperative to monitor the cognitive consequences in patients receiving active lipid-lowering therapies.

The advantages of our study were its large number of subjects from a prospective study, and conclusions based on longitudinal design. However, our study had several limitations. First, in our study, TC variability was calculated from the result of two blood tests (Wave 1 and Wave 3), while other studies had a larger number of blood tests ranging from 2 to 4. Second, 655 of 7,481 (8.76%) participants were excluded for the lack of cognition test results. The exclusion might have influenced the results. Third, the cognitive tests were limited. It could examine the major part of cognition. Considering that it was not a standardized test, there was no robust cut-off point for diagnosis of dementia, mild cognitive impairment, or significant cognitive decline.

The gender difference may be our new discovery. One previous study had not separated men and women, and viewed gender as a categorical variable when using MRLMs^10^. If we defined gender as a categorical confounder, we would have reached a similar conclusion: TC variability was significantly associated with cognition in Wave 3 through MRLMs (Supplementary Material 2). However, Tables 3 and 4 showed that the association did not exist in females.

There are some possible mechanisms to explain the gender difference. First, a previous study reported that decreases in serum TC increased the risk (OR 2.3) of dementia after approximately 20 years^12^. As the mean TC level in women was 0.3 mmol/L higher than that in men in our study (Supplementary Material 4), we hypothesized that the high TC levels protected women from the negative effects. Second, women had lower cognitive function than men. One previous study^17^, learning cognitive difference in CHARLS, attributed the cognitive difference to schooling, family and community levels of economic resource. Women might be tolerant to the impact of TC variability through unknown process.

## Conclusion

For men, individuals with higher visit-to-visit TC variability had lower cognitive function and suffered from a faster cognitive decline over a period of 4 years. The main domains showing decreases were memory, calculation, attention and orientation. The associations were not shown in women and need further research.

## Supplementary information

Supplementary information

## Data Availability

The data used in this manuscript from the China Health and retirement Longitudinal Study (CHARLS). We applied the permission for the data access (https://charls.pku.edu.cn/zh-CN) and got the access to use it. Prof. Yaohui Zhao (National School of Development of Peking University), John Strauss (University of Southern California), and Gonghuan Yang (Chinese Center for Disease Control and Prevention) are the principle investigators.
