# Supplementary material for: Higher visit-to-visit total cholesterol variability is associated with lower cognitive function among middle-aged and elderly Chinese men
Source: Sci Rep. 2020 Sep 23;10:15555. doi: 10.1038/s41598-020-72601-7 (PMC7511393; doi:10.1038/s41598-020-72601-7)
Supplement: Supplementary file 1 — Supplementary information [file 41598_2020_72601_MOESM1_ESM.docx]

Supplementary Material

Jianian Hua ^12#^, Yanan Qiao ^3#^, Chaofu Ke^3,^* and Yueping Shen^3,^**

^1^ Department of Neurology, The First Affiliated Hospital of Soochow University, Suzhou, Jiangsu, China.

^2^ Medical College of Soochow University, Suzhou 215123, PR China.

^3^Department of Epidemiology and Biostatistics, School of Public Heath, Medical College of Soochow University, 199 Renai Road, Suzhou, 215123, PR China.

^#^These authors contributed equally.

*** Correspondence:**Chaofu Ke
cfke@suda.edu.cn; Tel.: +86 512 6588 3323 (C.K.).

****Correspondence:**

Yueping Shen

shenyueping@suda.edu.cn; Tel.: +86 512 6588 3323 (Y.S.).

# Supplementary Material 1

Supplementary Material 1 showed the association between lipid-lowering therapy and cognitive scores in Wave 3 through MRLMs. Whether the individual was receiving lipid-lowering therapy was defined as a categorical variable (0=no, 1=yes). Males receiving lipid-lowering therapy had lower global cognition score in Wave 3 (*p*=0.037 for model1). The specific domain was figure drawing (*p*=0.021 for model 1).

Association between baseline lipid-lowering therapy and cognitive function in Wave 3

|  | Model 1 |  |  | Model 2 |  |
| --- | --- | --- | --- | --- | --- |
|  | Β (SD) | *p* |  | β (SD) | *p* |
| Male |  |  |  |  |  |
| Global cognition | -0.61 (0.29) | 0.037 |  | -0.41 (0.26) | 0.125 |
| Episodic memory | -0.12 (0.14) | 0.392 |  | -0.02 (0.13) | 0.575 |
| Figure drawing | -0.09 (0.04) | 0.021 |  | -0.09 (0.04) | 0.017 |
| TICS | -0.41 (0.21) | 0.054 |  | -0.27 (0.19) | 0.154 |
| Female | | | | | |
| Global cognition | 0.18(0.25) | 0.470 |  | 0.13(0.22) | 0.556 |
| Episodic memory | -0.07(0.11) | 0.546 |  | -0.08(0.11) | 0.449 |
| Figure drawing | 0.00(0.03) | 0.926 |  | 0.00(0.03) | 0.993 |
| TICS | 0.25(0.18) | 0.172 |  | 0.23(0.16) | 0.166 |

Model1: adjusted for age, mean TC, education, marital, residential area, BMI, smoking, drinking, depression, hypertension, dyslipidemia, diabetes mellitus, stroke, heart disease and cancer

Model2: Model 1 + baseline cognition

# Supplementary Material 2

Supplementary Material 2 showed the association between TC variability and cognitive scores in Wave 3 through MRLMs. Gender was defined as a categorical variable (1=male, 2=female). The following table showed the beta coefficient of TC variability and gender in the MRLMs.

Association between TC variability and cognitive function in Wave 3

|  | TC Variability |  |  | Gender |  |
| --- | --- | --- | --- | --- | --- |
|  | β (SD) | *P* |  | β (SD) | *P* |
| Model 1 |  |  |  |  |  |
| Global cognition | -0.38 (0.15) | 0.012 |  | -0.84 (0.14) | <0.001 |
| Episodic memory | -0.09 (0.07) | 0.175 |  | 0.09 (0.06) | 0.161 |
| Figure drawing | -0.02 (0.02) | 0.233 |  | -0.09 (0.02) | <0.001 |
| TICS | -0.27 (0.11) | 0.016 |  | -0.84 (0.11) | <0.001 |
| Model 2 | | | | | |
| Global cognition | -0.37 (0.14) | 0.006 |  | -0.29 (0.12) | 0.017 |
| Episodic memory | -0.11 (0.07) | 0.088 |  | 0.09 (0.06) | 0.123 |
| Figure drawing | -0.02 (0.02) | 0.304 |  | -0.06 (0.02) | <0.001 |
| TICS | -0.24 (0.10) | 0.018 |  | -0.39 (0.09) | <0.001 |

Model1: adjusted for gender, age, mean TC, education, marital, residential area, BMI, smoking, drinking, depression, lipid-lowering therapy, hypertension, dyslipidemia, diabetes mellitus, stroke, heart disease and cancer

Model2: Model 1 + baseline cognition

# Supplementary Material 3

Supplementary Material 3 showed the relationship between variability of other metabolic parameters and cognitive scores in Wave 3. The beta coefficients of HDL, Glu, TG and SBP were all close to zero.

Association between variability in other metabolic parameters and cognitive function in Wave 3*

|  | Model 1 |  |
| --- | --- | --- |
|  | β (SD) | *P* |
| Male | | |
| LDL | -0.28 (0.25) | 0.269 |
| HDL | -0.02 (0.02) | 0.133 |
| Glu | -0.01 (0.01) | 0.248 |
| TG | -0.00 (0.00) | 0.140 |
| SBP | -0.00 (0.00) | 0.094 |
| Female | | |
| LDL | 0.34 (0.23) | 0.140 |
| HDL | -0.01 (0.02) | 0.549 |
| Glu | -0.01 (0.01) | 0.018 |
| TG | -0.00 (0.00) | 0.349 |
| SBP | 0.01 (0.00) | 0.098 |

Abbreviations: LDL: low density lipoprotein; HDL: high density lipoprotein; Glu: glucose; TG: trygliceride; SBP: systolic blood pressure.

*Adjusted for age, mean TC, education, marital, residential area, BMI, smoking, drinking, depression, lipid-lowering therapy, hypertension, dyslipidemia, diabetes mellitus, stroke, heart disease and cancer

# Supplementary Material 4

Supplementary Material 4 showed the sex difference in health factors (Wave 1) and TC variability. We used ANOVA or the Pearson χ2 test

Baseline characteristics between men and women

|  | Male  (n=2892) | Female  (n=3480) | *P-value* |
| --- | --- | --- | --- |
| Continuous Variables |  |  |  |
| Age, y | 59.7(8.6) | 57.4(8.7) | <0.001 |
| Mean TC, mmol/L | 4.7(0.8) | 5.0(0.9) | <0.001 |
| TC variability | 0.3(0.6) | 0.3(0.6) | 0.737 |
| Categorical Variables |  |  |  |
| Education |  |  | <0.001 |
| Illiterate | 333(11.5) | 1328(38.2) |  |
| Primary school | 1348(47.9) | 1357(39.0) |  |
| Middle school | 779(27.0) | 558(16.0) |  |
| High school and above | 395(13.7) | 236(6.8) |  |
| Marital status |  |  | <0.001 |
| Married | 2674(92.4) | 3074(88.3) |  |
| Other status | 219(7.6) | 406(11.7) |  |
| Residential area |  |  | 0.009 |
| Urban | 435(15.1) | 609(17.5) |  |
| Rural | 2452(84.9) | 2867(82.5) |  |
| Depression |  |  | 0.425 |
| Yes | 193(6.7) | 250(7.2) |  |
| No | 2699(93.3) | 3230(92.8) |  |
| BMI (kg/m^2^) |  |  | <0.001 |
| <18.5 | 231(8.0) | 231(6.6) |  |
| 18.5-28 | 2392(82.7) | 2673(76.8) |  |
| >28 | 269(9.3) | 576(16.6) |  |
| Current smoker | 2327 (80.5) | 285(8.2) | <0.001 |
| Current drinker | 1326(45.9) | 279(8.0) | <0.001 |
| Hypertension | 733(25.4) | 991(28.5) | 0.005 |
| Dyslipidaemia | 311(10.8) | 427(12.3) | 0.060 |
| Diabetes mellitus | 163(5.6) | 253(7.3) | 0.009 |
| History of heart disease | 329(11.4) | 538(15.5) | <0.001 |
| History of stroke | 68(2.4) | 66(1.9) | 0.208 |
| Lipid-lowering therapy | 168(5.8) | 275(7.9) | 0.001 |

# Supplementary Material 5

Supplementary Material 5 showed the effect of increasing or decreasing TC level separately, using multivariate linear regression models.

Association between increasing or decreasing TC level and cognitive function in Wave 3

| Decrease | | | | | |
| --- | --- | --- | --- | --- | --- |
|  | Model 1 |  |  | Model 2 |  |
|  | β (SD) | *P* |  | β (SD) | *P* |
| Male (n=1836 from 2897) | | | | | |
| Global cognition | -0.88 (0.25) | <0.001 |  | -0.76 (0.22) | <0.001 |
| Episodic memory | -0.30 (0.12) | 0.009 |  | -0.29 (0.11) | 0.009 |
| Picture | -0.04 (0.03) | 0.191 |  | -0.04 (0.03) | 0.205 |
| TICS | -0.53 (0.18) | 0.003 |  | -0.44 (0.17) | 0.008 |
| Female (n=2018 from 3485) | | | | | |
| Global cognition | -0.12 (0.29) | 0.645 |  | -0.14 (0.25) | 0.582 |
| Episodic memory | 0.05 (0.13) | 0.712 |  | 0.07 (0.13) | 0.580 |
| Picture | 0.00 (0.04) | 0.898 |  | 0.01 (0.03) | 0.820 |
| TICS | -0.18 (0.21) | 0.367 |  | -0.23 (0.18) | 0.212 |
| Increase | | | | | |
|  | Model 1 |  |  | Model 2 |  |
|  | β (SD) | *P* |  | β (SD) | *P* |
| Male (n=1054 from 2897) | | | | | |
| Global cognition | 0.10 (0.21) | 0.651 |  | 0.16 (0.29) | 0.410 |
| Episodic memory | 0.00 (0.10) | 1.000 |  | 0.05 (0.09) | 0.617 |
| Picture | 0.03 (0.21) | 0.213 |  | 0.03 (0.03) | 0.263 |
| TICS | 0.06 (0.15) | 0.692 |  | 0.05 (0.14) | 0.69 |
| Female (n=2018 from 3485) | | | | | |
| Global cognition | -0.01 (0.17) | 0.943 |  | 0.03 (0.15) | 0.858 |
| Episodic memory | 0.01 (0.08) | 0.873 |  | 0.05 (0.07) | 0.451 |
| Picture | -0.01 (0.02) | 0.700 |  | -0.01 (0.02) | 0.606 |
| TICS | -0.02(0.13) | 0.897 |  | -0.04 (0.11) | 0.751 |

Model1: adjusted for age, mean TC, education, marital, residential area, BMI, smoking, drinking, depression, lipid-lowering therapy, hypertension, dyslipidemia, diabetes mellitus, stroke, heart disease and cancer

Model2: Model 1 + baseline cognition
